# Nurse-patient relationship boundaries and power: A critical discursive analysis

**DOI:** 10.1177/09697330251314093

**Published:** 2025-01-19

**Authors:** Jeanette Varpen Unhjem, Marit Helene Hem

**Affiliations:** 5562 Molde University College; 570217 NTNU Social Research; 87368 VID Specialized University

**Keywords:** nurse-patient relationship, professional boundaries, professional ethics, qualitative research, discourse analysis

## Abstract

**Introduction:** Mental health nursing is dependent on nurses’ ability to engage in therapeutic relationships with patients. The ability to manage professional boundaries is equally important, but less explored. This study aims to address the following research questions: How do nurses define their professional, personal, and private roles? What are nurses’ experiences with professional boundaries? What are the implications of nurses’ understanding of these boundaries?

**Background:** Nurse–patient relationships are characterized by asymmetrical power dynamics, which places the responsibility of delineating professional boundaries on the nurse. While ethical codes offer guidance, nurses must identify appropriate boundaries in a dynamic process that relies on the clinical context.

**Research design:** This study used critical discursive psychological analysis to examine data from participant observations, individual interviews, and focus group discussions.

**Participants and research context:** We included 16 nurses working in mental health care in this study, comprising 12 in specialist mental health care and 4 in community mental health care.

**Ethical considerations:** The study was registered with Norwegian Social Science Data Services.

**Results:** Nurses defined professionalism as being an educated caregiver who prioritizes patients’ needs. Professionalism involves personal engagement, while some personal matters remain private. However, nurses experienced challenges in maintaining professional boundaries, facing dilemmas due to the subjective nature of boundaries and patients’ unpredictable responses.

**Conclusions:** While nurses prioritize patients’ needs and best interests, this study demonstrated that personal engagement is considered part of professionalism. However, nurses encounter complex dilemmas in setting professional boundaries. Additionally, these boundaries can either emphasize or de-emphasize the power differential in nurse-patient relationships.

## Introduction

In mental health nursing practice, the ability to establish therapeutic relationships with patients is a fundamental competence. Professional boundaries refer to the appropriate behaviors expected in therapeutic relationships.^1^ Studies on nurse-patient relationships often address how to build therapeutic relationships, while the issues of boundaries are explored to a lesser extent.

This study aimed to explore nurses’ understanding of professional boundaries using discourse analysis to examine how they talk about being professional, personal, and private in relationships with patients in mental health care. We posit that analyzing how nurses perceive and discuss professional boundaries can reveal the complexities and dilemmas they encounter in nurse–patient relationships. This knowledge may help identify important challenges that nurses experience in mental health care.

The objective of this study was to address the following research questions: How do nurses define being professional, personal, and private? What are nurses’ experiences with practicing professional boundaries? What are the implications of nurses’ understanding of professional boundaries?

## Background

Therapeutic relationships are interpersonal processes in which nurses work together with patients to meet their health needs.^2,3^ These relationships are characterized by significant knowing and meaningful connectedness.^4^ According to the International Council of Nurses,^5^ a professional relationship between nurses and patients is “an ongoing interaction between two people that observes a set of established boundaries or limits that is deemed appropriate under governing ethical standards.”

The inherent power imbalance, stemming from nurses’ authority and patients’ vulnerability, necessitates clear professional boundaries to uphold patient-centered care.^3^ Nurses must see themselves as separate from the patients and keep an exclusive focus on their needs, interests and concerns.^3^ It is the duty of the nurse as a healthcare professional to maintain these boundaries,^3^ and setting boundaries is a crucial competency in mental health nursing,^6^ partly because it facilitates healthy independence.^7^

Suryani et al.^8^ identified that nurses’ understanding of professional boundaries encompasses defining these boundaries, understanding their goals and benefits, identifying the areas where boundaries are set, and recognizing factors that support and challenge boundary setting. Mental health professionals manage boundaries through a dynamic and evolving process,^9^ in which there is gradual transition from an appropriate therapeutic relationship to unprofessional behaviors such as under- or over-involvement.^3^ Gutheil and Gabbard noted that while the concept of professional boundaries is often understood instinctively, it is challenging to explain or practice in clinical settings,^10^ as appropriate boundaries rely on judgment and interpretations related to the context of the therapeutic relationship.^1,11^ The flexibility of professional boundaries can make it difficult to identify concrete guidelines.^12^ Some advocate tools, while others argue that ethical guidelines, codes of practice, and standards can inform but not determine boundary decisions in clinical practice.^13^

Given the lack of explicit guidelines, nurses must independently manage professional boundaries in clinical settings, balancing close therapeutic relationships with the risk of disciplinary actions if boundaries are not appropriately set. Disciplinary boards often operate with a narrow scope regarding nurse–patient relationships, meaning that boundary transgressions can lead to professional misconduct and disciplinary actions.^14^ Boundary violations are a common reason for disciplinary actions against nurses, and comprise non-sexual boundary violations as well as sexual misconduct.^15^ Research on disciplinary actions taken by healthcare regulatory bodies frequently focuses on nurses, making them the second most studied profession in this context,^16^ and highlighting the importance of this issue in nursing practice. There is, however, not much research on nurses’ experiences with the management of professional boundaries.

Professional boundaries are based on the premise that nurse–patient relationships are inherently asymmetrical in terms of power, favoring the nurse. Thus, nurses bear the responsibility and the power to maintain these boundaries.^3^ Understanding power in the context of nurse-patient relationships is essential because nurses are uniquely positioned to empower patients.^17^ Power can be defined in various ways, depending on the theoretical framework and context. It can be considered as an intrapersonal construct possessed by an individual or as something that is exercised in interpersonal relationships.^18^ In this study, power is considered as the ability to define professional boundaries in interpersonal relationships between nurses and patients.

Discourse analysis is a broad theoretical framework rather than a specific analytic method.^19^ It focuses on patterns in people’s talk and the functions and consequences of that talk.^19^ In discursive psychology, written and spoken language are viewed as constructions of the world oriented towards social action.^20^ According to discursive psychology, language is not merely an expression of experiences but actively constructs experiences and subjective, psychological reality. Thus, language constructs social reality rather than merely reflecting it. Critical discursive psychology is one possible approach within discourse methodologies.^21^ It offers a unique analytic perspective by combining attention to the micro-level elements of language with the macro-level elements of context.^21^ By applying this dual reading of the material, we can articulate how nurses construct professional boundaries both linguistically and in practice.

## Methods

A qualitative approach was employed to explore the complex issues of professionalism, personal engagement, and privacy in nurse-patient relationships in mental healthcare. This approach was deemed appropriate to gain insights into nurses’ thoughts, experiences, and actions concerning these concepts. Moreover, qualitative methods are preferable for achieving a comprehensive understanding of complex issues.^22^ We employed source triangulation, integrating participant observations, individual interviews, and focus group discussions. Consistent with the emergent character of qualitative research,^22^ data from these various sources were interdependent: insights from participant observations inform the development of interview guides, while findings from interviews guided the construction of a vignette used to stimulate focus group discussions.^23^

The trustworthiness of the study was ensured by using criteria such as credibility, transferability, dependability, and confirmability.^24^ Credibility was warranted by triangulation in the data collection process: Multiple data sources were used to validate findings by integrating participant observations, individual interviews, and focus group discussions. Further, the data collection process spanned nine months, allowing for an in-depth understanding of the nurses’ experiences and ensuring that the findings were adequately grounded in their context. The primary author conducted the interviews and transcribed the data, allowing for a deep and prolonged engagement with the material, which enhances the credibility of the findings. Transferability was safeguarded by thick descriptions of the participants’ backgrounds, the research context, and the analytic process, providing a rich basis for understanding the findings and assessing their applicability to other settings. In addition, the use of purposeful sampling ensured that a diverse range of experiences and perspectives were included. Dependability was maintained by detailed records of the data collection process, including the duration and nature of observations, interviews, and focus group discussions. Confirmability was ensured secured by the primary author through the maintenance of a reflexive journal to document potential biases and reflections throughout the research process, ensuring that the findings were shaped by the data rather than personal biases.

### Participants and research context

A purposeful sampling strategy was employed, involving 16 mental healthcare nurses. Requests for participation in the study were sent to a selection of specialist and community mental health services, which in turn provided contact information on potential participants. The first author followed up with on-site information during staff meetings, and recruited additional participants. Participants comprised 13 women and 3 men, aged between 40 and 60 years (mean = 52 years, median = 54.5 years). All participants were registered nurses, with most (*n* = 15) specializing in mental healthcare. The participants had between 5 and 37 years of nursing experience (mean = 21 years, median = 22 years). The sampling strategy aimed to ensure site-level variation, including 12 nurses from four different psychiatric units in specialist mental health care, and four from three community mental health districts in Western Norway. A combination of convenience and snowball sampling techniques was employed to achieve the desired sample size.

### Data collection

Data collection took place over 9 months, from July 2013 to March 2014. The process involved two rounds of participant observation sessions (with one nurse attending three sessions), multiple individual interviews (typically two interviews, although three of the nurses underwent three sessions), and focus group discussions (three nurses were unable to participate). Participant observations aimed to identify the types of personal information shared by nurses with patients. Individual interviews ranged from 46 min to 1 h and 47 min, and covered various topics including nurses’ perceptions of being professional, personal, and private. Focus group discussions delved into the exploration of professional boundaries, lasting between 1 h and 52 min to 2 h. Two focus group interviews involved five participants each, while one included three participants.

### Ethical considerations

An application was submitted to the regional ethics committee, which determined in March 2013 that the study fell outside the scope of the Norwegian Health Research Act, obviating the need for approval. The project was duly registered with the Norwegian Social Science Data Services, as required for all research projects handling personal data in Norway. Participants received detailed verbal and written information about the study’s objectives and methodologies before providing written consent. Rigorous measures were implemented to maintain confidentiality during data transcription and publication, safeguarding participants’ anonymity.

### Data analysis

We employed critical discursive psychology, focusing on three key analytic concepts: interpretative repertoires, ideological dilemmas, and subject positions.^25^ Edley described interpretative repertoires as “relatively coherent ways of talking about objects and events in the world.”^25^ These repertoires are rooted in common sense and historical context, influencing how people discuss and perceive phenomena. Common sense and historical context make up the macro-level elements of critical discursive psychology. Ideological dilemmas arise from competing or contradictory interpretative repertoires concerning the same phenomenon.^25^ Subject positions refer to identities relevant to specific ways of talking.^25^ The micro-level elements of wording and interactions become conduits for the macro-level elements. Edley emphasizes the importance of familiarity with the data in recognizing interpretative repertoires, suggesting that the data collector often has an advantage in this regard.^25^ The primary author conducted the interviews and transcribed the data, facilitating a deep understanding of the material over years of analysis.

Subsequently, the data were imported and coded using NVivo12. Passages discussing professional boundaries, whether professional, personal, or private, were identified and analyzed. Sentences containing terms such as “professional,” “personal,” or “private” were coded, along with their surrounding context. We then explored the dataset by asking the following three analytical questions, as suggested by Edley: What interpretative repertoires are evident? What evidence indicates ideological dilemmas? What subject positions do participants adopt?^25^ We coded the text that answered these questions, grouped text passages and gave the groups preliminary titles. Groups of text passages were then re-examined and re-ordered, until three distinct interpretative repertoires emerged from the data. Additionally, two ideological dilemmas and two subject positions closely connected to interpretative repertoires were identified. Consistent with our findings, Edley also noted the interconnectedness between ideological dilemmas and interpretative repertoires, as both shape social interaction and thought.^25^ Subject positions are inferred from discourse patterns, reflecting the ways participants position themselves within the discussion.

## Results

### Interpretative repertoires

#### Prioritizing patients’ needs

As per the nurses’ accounts, prioritizing patients’ needs was deemed essential for professionalism. The nurses discussed two distinct approaches to prioritizing patients’ needs. Firstly, nurses emphasized the significance of being attentive to patients’ needs and directing their focus towards the patients rather than themselves. Secondly, nurses described adjusting professional boundaries based on what they perceived to be in the patient’s best interests.

A patient-centric approach was linked to an understanding that many patients already have challenges in their lives and may not desire involvement in nurses’ personal matters. One nurse mentioned that patients may need “peace” and could become “irritated” if the conversation revolves too much around nurses’ personal lives. As a result, nurses often assessed whether patients were “receptive” to receiving their personal information, relying on their intuition to “sense” if the patient was open to listening. The nurses consistently emphasized maintaining focus on the patients. One nurse expressed, “Some patients may be curious about us, but in a conversation, if the focus is more on me than on the patient, that would be completely wrong.” Other phrases such as “prioritizing others’ needs over my own” were common in the nurses’ discussions about focusing on patients rather than themselves. One nurse stated, “I am here for the patients,” and another framed it as “not sharing everything that comes to mind,” as “we have to be professional and helpful.”

Adjusting professional boundaries in the patient’s best interests was described as using personal information “purposefully,” “conscientiously,” or “constructively.” Nurses viewed sharing personal information with patients to be “useful” or “beneficial” in some ways. For example, one nurse recounted how she shared a story about her parental anxiety to assist a patient dealing with anxiety. The nurse explained how she used cognitive techniques to manage her concerns, hoping her narrative could offer the patient a perspective to make similar choices in managing anxiety. The patient later expressed gratitude, mentioning that the nurse’s story had been helpful in her situation.

#### Incorporating personalization into professionalism

The main connection between professionalism and being personal lies in the acknowledgment that being personal is an integral component of being professional. Professionalism devoid of the nurses’ personal engagement represents a diminished form of professionalism, resulting in poor nurse-patient relationships.

Integrating personal elements into professional interactions was seen as enhancing the nurse-patient relationship: By being personal, the nurse often received a deeper connection in return. “I am more interested in getting to know the person than just focusing on diagnoses,” expressed one nurse. “If a patient is passionate about horses, animals, nature, or travel, I find it important to share some of my own experiences in those areas.” Conversely, maintaining a strictly impersonal demeanor could negatively impact the nurse-patient relationship. The nurse suggested that if she remained completely detached and only listened without sharing anything personal, it would hinder the development of a meaningful relationship with the patient, as interactions with nurses who do not disclose personal details may be less engaging.

When questioned about the possibility of performing nursing duties effectively without incorporating personal elements, one nurse expressed uncertainty about its feasibility. “Acting in a completely impersonal manner simply would not work,” explained another nurse, emphasizing the importance of establishing a sense of trust that the nurse can provide assistance. Furthermore, the nurses regarded certain qualities such as empathy as essential components of nursing that encompass both professional and personal dimensions. Thus, “it is part of being professional to have empathy, but one must be able to connect with the patient’s emotions and personal side to do so effectively.”

#### Maintaining privacy

Respecting privacy entailed refraining from disclosing information related to illnesses, personal issues, or family matters. It involved sharing life experiences in a manner that extended beyond casual anecdotes for the patient’s benefit. This could encompass details that nurses choose not to divulge to anyone, including patients or others. One nurse explained, “There is an inner circle of my closest friends and family who know all about me, but only I know everything about myself. So, I keep it very private.”

Being private within nurse-patient relationships implied sharing details that might not be disclosed to individuals beyond close friends. Being private in these relationships suggested a level of intimacy typically reserved for friendships. A nurse emphasized the distinction by stating, “You have friends that you can share these things with instead of with patients,” indicating that relationships with friends and family are notably different from relationships with patients. Another one expressed “I am not here at work to share my problems.” In her opinion, it would be ethically inappropriate to discuss her problems with patients.

The prevailing sentiment among nurses regarding privacy was that private information was generally not shared with patients. However, some nurses recounted instances when they did share private experiences with patients. For example, one nurse mentioned “I talk about my children and perhaps some private things.” Nonetheless, the decision to disclose private information to patients was sometimes influenced by the nurses’ self-perception. For instance, one nurse remarked “I find it challenging because it reflects my approach towards patients.” Similarly, while sharing private experiences was considered a part of the nurse’s demeanor, it was contingent on the specific nurse-patient relationships. One nurse explained “I am not the same with all patients. I do not have the same connections with every patient. As a result, it is not natural for me to divulge such details to every patient.” Moreover, there were specific situations where nurses chose to share private experiences, believing they could benefit the patient. For instance, despite one nurse stating that “you are supposed to keep a certain distance,” she provided examples of cases where they had made exceptions based on the context and their judgment. Some nurses found that community care settings would make them particularly attentive to maintaining a professional role.

### Ideological dilemmas

#### Boundaries are subjective

Participants consistently indicated that professional boundaries are subjective and depend on individual deliberations and decision-making. One nurse reflected that “It becomes a boundary that each and everyone must make up themselves.” Another nurse mentioned that “I have perhaps opened up more and more,” noting uncertainty about boundaries compared to those of other nurses. She concluded, “I have my interpretation of it. Which is what I must be comfortable with. And what others share, they must be comfortable with because it is not my concern.”

The subjectivity of professional boundaries creates ideological dilemmas in two ways:

(i) Self-defined boundaries: Nurses had to explore and establish their professional boundaries independently, without guidance from colleagues or standardized guidelines, even when new to mental healthcare settings. One nurse recounted, “When I began working here, I had never worked in mental healthcare settings. I was set to take care of those with eating disorders who were boundaryless. I had no recipes or templates to follow. Many of my colleagues have worked for more than 20 years and are skilled. I had to figure it out myself.”
(ii) Conflict and critique: Differences in boundary settings among nurses could lead to open or hidden critiques and conflicts. For instance, in one psychiatric ward, a debate arose over whether a staff sign-up list for an expensive holiday party should be visible to patients. One nurse believed the information was private and should not be visible to patients, many of whom had limited financial resources. Other nurses argued that removing the list was unnecessary and that it was a way of showing the patients that the staff “were humans too.”

Similarly, nurses occasionally felt that colleagues crossed their boundaries. For example, one nurse, despite being generally open, expressed discomfort when colleagues shared what she deemed unnecessary information with patients: “Even if I am an open person myself, […] I have had those moments where I think that some things were not necessary to talk about with patients.” Nonetheless, although nurses sometimes manage boundaries differently, they emphasize trusting their colleagues’ boundary decisions: “Sometimes, patients and nurses find that they have much in common, and it is natural that they develop a close relationship. It can seem too close to others, but one must trust that a professional is capable of managing their boundaries.”

#### Unpredictability of patients’ responses

Nurses recognized the necessity of being cautious when discussing personal topics or establishing relationships with certain patient groups due to the unpredictability of their responses. Sensitive topics included money, politics, family relations, and travel, which nurses often avoided. One nurse noted that “we do not always understand everything – how a person reacts,” emphasizing that it requires time to build an understanding of individual patients. Despite this, she noted the potential value in “daring to make some mistakes too,” acknowledging that being personal could benefit the nurse-patient relationship, even if it involved taking some risks.

Some nurses recounted negative experiences where patients reacted poorly to their personal stories. For instance, one nurse shared her experience of grief with a patient, hoping to express empathy, but the patient felt offended. The nurse reflected, “it became a competition about who was worse off. I completely miscalculated and thought I knew her better. I learned a lot from that experience. You have to be very careful about using private examples because you do not know how well it will be received.”

If a nurse crosses a patient’s boundaries, it could create a sense of “discomfort” or of “something being wrong.” One nurse described feeling uneasy after briefly confiding in a patient, realizing afterward that “this was wrong.” These boundaries were often felt intuitively rather than consciously assessed. Sometimes, the sensation of boundary crossing became apparent only through the nurse’s emotional reactions or an unexpected negative response from the patient. Consequently, nurses referred to appropriate professional boundaries as “being balanced,” where the nurses’ openness and professional distance were adjusted to the specific context and felt comfortable for both the nurse and the patient.

In addition to being sensitive to patients’ financial hardships, nurses were cautious about sharing personal information out of concern for their own and their colleagues’ safety. One nurse stated, “We do have patients here who, in my experience, we do not share anything at all with, not where you live or anything private.” This caution was particularly heightened when dealing with patients diagnosed with psychiatric conditions like antisocial personality disorders. The nurse elaborated, “you do not want such persons to suddenly be at your door at home.” The potential risk of patients invading their privacy, perhaps violently, made nurses particularly apprehensive about sharing personal information and experiences.

### Subject positions

When nurses discussed their professional roles, two distinct subject positions emerged. The first subject’s position portrayed nurses as educated helpers closely associated with prioritizing patients’ needs, aligning with the interpretative repertoire. Through this position, nurses established clear roles and responsibilities, emphasizing the importance of placing patients’ well-being at the forefront. Additionally, nurses also conveyed a secondary subject position: that of the nurse as the patients’ fellow human being. This complementary position, supported by the interpretative repertoire, highlighted the significance of personal connections in professionalism.

#### The nurse as an educated helper

When discussing their professional identity, nurses emphasized their role as educated helpers. Education encompassed graduating from nursing school or pursuing further education, along with acquiring professional knowledge. One nurse succinctly expressed, “Being professional means I am content with the knowledge and experiences that I have gained, particularly regarding patients’ issues here.” Some nurses stressed the importance of staying updated with field advancements. For instance, one nurse stayed informed through web searches and reading nonfiction books. She emphasized that “what was applicable 20 years ago may not hold true today.”

Moreover, being educated entailed maintaining a professional demeanor in patient care. A nurse articulated, “being professional involves maintaining a poised outlook while also leveraging our expertise in building relationships.” The capacity to cultivate therapeutic relationships with patients is a hallmark of professionalism developed through education and past experiences. Despite the significance of personal qualities, education remains indispensable, positioning nurses as the most knowledgeable professional caregivers.

#### The nurse as a fellow human being

While the focus was on their role as educated helpers, nurses also portrayed themselves as fellow human beings to patients. This was evident through phrases like “equal” and “fellow man.” When using the term “medmenneske.” a Norwegian word for fellow man, nurses highlighted a role alongside their professional identity. One nurse described her workday as encompassing various situations, where she alternated between identifying as a psychiatric nurse or simply a fellow human being. These roles shifted based on whether it was a treatment scenario or an everyday situation.

However, equality did not exempt nurses from their professional duties. One nurse concluded, “despite our equality, I am still the helper.” Thus, the nurse’s role as a fellow human being complemented their professional identity as educated helpers. Ultimately, the professional role encompassed being both a person and an expert caregiver.

## Discussion

This study aimed to address the following research questions. How do nurses define themselves as professional, personal, and private? What are nurses’ experiences of practicing professional boundaries? What are the implications of nurses’ understanding of professional boundaries? Through critical discursive psychological analysis, we discovered that nurses define professionalism as embodying an educated helper who prioritizes patients’ needs. Professionalism entails being personal, although certain personal matters remain private. When discussing the practice of professional boundaries, nurses highlighted significant challenges, including dilemmas related to the subjective nature of boundaries and the unpredictability of patients’ responses. Furthermore, aligned with critical discursive psychological analysis, we examine the implications of nurses’ understanding of professional boundaries. Given the fundamental role of power and vulnerability in nurse–patient relationships, particularly in mental health nursing,^26^ we focused on how power dynamics are influenced by the discursive structures identified in this study.

The power imbalance in nurse–patient relationships is particularly pronounced in mental health care,^26^ where overt coercive practices serve as evident expressions of power. In contrast, making decisions regarding patients’ best interests represents a less visible form of power – a hidden or invisible expression.^27^ In this study, nurses emphasized prioritizing patients’ needs when navigating professional boundaries. Consequently, nurses should establish appropriate boundaries based on professional assessments of patients’ needs.^3^ Nurse interventions and treatment should stem from their evaluations of what serves the best interest of the patients, granting nurses the power to determine the type of assistance that patients receive. Although patients’ needs can be perceived as powerful, given their precedence in shaping nurse–patient relationships, nurses ultimately wield the authority to determine the significance of various needs. Moreover, deciding what will be and what will not be addressed is another form of hidden or invisible power.^27^

Recognizing and maintaining relationship boundaries is an integral part of the professional role of the nurse.^5^ Boundaries in the nurse-patient relationship are deemed inappropriate if they stem from nurses’ needs or lack professional rationale.^3^ This study underscores how nurses prioritize focusing on patients rather than themselves as a hallmark of professionalism. In the nurse–patient relationship, the patient assumes the central role, leading nurses to consider certain topics, such as family matters or personal illness, as private and not shared with patients. However, some mental health nurses consider sharing personal information that has no therapeutic value for the patient as a minor boundary crossing.^9^ While nurses’ boundaries often center on patient consideration, they also wield power to define what is private. This authority to determine discussion topics exemplifies another form of hidden or invisible power.^27^

This study also revealed that nurses view being personal, including the sharing of personal information, as an essential aspect of professionalism. Being personal is crucial for nurturing supportive relationships.^12^ Nurses cannot and should not adopt a completely impersonal stance in the nurse-patient relationship. Instead, they should strive to strike a balance between personal needs and prioritizing patients’ needs. However, nurses frequently encounter situations where the professional boundaries of the nurse-patient relationship are tested.^6^ Regardless of this, nurses hold positions of power in the relationship, partly due to their access to patients’ intimate information.^3^ While sharing personal information can serve as a tool to mitigate information asymmetry and reduce the power imbalance in the relationship, differing opinions between nurses and patients regarding what should be kept private can arise. Some patients may be perceived as overly inquisitive by nurses, while some nurses may be viewed as overly secretive by patients. Thus, withholding personal information can be perceived as a power play, highlighting the information asymmetry. Conversely, it may indicate that nurses feel powerless and need to protect themselves. Ideally, boundaries should be mutually understood,^11^ and patients should be aware of appropriate roles and behaviors in therapeutic relationships.^12^

In this study, nurses described two key dilemmas complicating boundary setting: the subjectivity of professional boundaries and the unpredictability of patients’ responses. Nurses experienced boundaries as highly subjective, necessitating individualized identification of appropriate limits while managing differing perspectives among colleagues. This subjectivity is compounded by a lack of discussion on boundary issues during their formative educational programs,^11^ contributing to insufficient guidance and appropriate guidelines in clinical settings. Additionally, the unpredictability of patients’ responses to boundaries makes nurses cautious about personal engagement, even though self-disclosure is common among them.^23^ Despite these issues, nurses are expected to discern and act correctly, even in situations where it is unclear what the right thing is.^6^

The inherent subjectivity and unpredictability make it nearly impossible for nurses to define appropriate boundaries. Concrete or fixed boundaries are challenging to establish, as they must be deliberated and defined based on individual patients and situations.^12^ This relativity affords nurses significant power in choosing which boundaries to set, when, and with whom. In a previous paper, we discussed the phenomenon of dual relationships, meaning relationships with patients that are pursued outside of working hours.^28^ We showed that dual relationship were complex and highly contextually dependent, and that the nurses displayed strong ambivalence in these relationships. Appropriate boundaries depend on the specific nurse-patient relationships, clinical setting, and broader context.^9^ Although patients’ responses might challenge the boundaries set by nurses, patients do not have the power to dictate professional boundaries. Consequently, the hidden or invisible nature of this power to decide what is addressed or what is in the patient’s best interests can lead to an underestimation of nurses’ power. Furthermore, professional boundaries may evolve over time as therapeutic relationships progress and deepen,^9^ necessitating ongoing negotiation within nurse-patient relationships.

Another interesting finding of this study is how nurses portrayed themselves as the patients’ fellow human beings. People with severe mental illness describe the experience of shared humanness as an essential and helpful component of relationships with professionals.^12^ The position of a fellow human being suggests equality between patients and nurses, in contrast with the inherently asymmetrical view of the nurse-patient relationship. The relationship can be experienced as characterized by connectedness and reciprocity.^4^ Nurses’ depiction of themselves as the patients’ fellow human beings may indicate a lack of acknowledgment of the power differential between nurses and patients in therapeutic relationships in the context of mental health care. As power dynamics can be hidden or invisible, this notion of being a patient’s fellow human being can further obscure the power differential. Further research could examine how changes in patient legal rights and the emphasis on patient empowerment affect therapeutic relationships and the management of professional boundaries by the nurses. Moreover, nurses positioning themselves alongside patients as equals can be considered as a reaction to the power imbalance, thereby seeking to diminish their own power. Conversely, Pieranunzi^17^ found that nurses felt most powerful in relationships where they viewed themselves as deeply connected and personal, transcending their roles and relating to the humanness of the patient.

## Limitations

Given that the empirical data was gathered 10 years ago, it is reasonable to question their relevance today. However, based on our extensive experience in the field and understanding of nursing practice, we contend that the interview data remain valid for several reasons. First, the study participants were recruited from hospital wards and community mental health facilities. Over the past decades, these areas have not seen any remarkable changes in terms of organization, patient demographics, or operational methods. Second, achieving a balance between being professional, personal, and private remains a fundamental principle in mental health nursing, essential for upholding ethical and professional standards. Lastly, this topic remains highly relevant, as media coverage frequently highlights cases where health professionals are criticized for failing to maintain appropriate boundaries with patients. Boundary violations continue to be a significant cause of disciplinary actions against nurses, both in Norway and internationally, highlighting the importance of research aimed at enhancing the understanding of the complex professional boundaries that nurses must navigate to provide effective patient care.

## Conclusion

This study explored how nurses discuss and practice professional boundaries with mental healthcare patients. While nurses prioritize patients’ needs and best interests, our findings highlighted that they also consider being personal as part of professionalism. However, nurses encountered dilemmas in maintaining these boundaries. Additionally, the practice of professional boundaries exemplifies hidden or invisible power, where these boundaries can either emphasize or deemphasize the inherent power differential in nurse-patient relationships. Further research should investigate the connection between professional boundaries, moral stress, and burnout. Ethical reflection may also provide strategies for nurses to navigate boundary setting more effectively, and including patient perspectives, as well as those of peer workers and volunteers, in future studies would be valuable. Moreover, developing context-sensitive guidelines for professional boundary setting could contribute to improved practice standards in mental healthcare.
